# Supplementary material for: Development and evaluation of habitat suitability models for nesting white-headed woodpecker (Dryobates albolarvatus) in burned forest
Source: PLoS One. 2020 May 15;15(5):e0233043. doi: 10.1371/journal.pone.0233043 (PMC7228071; doi:10.1371/journal.pone.0233043)
Supplement: S1 Appendix — (DOCX) [file pone.0233043.s001.docx]

**S1 Appendix.** Plot sizes for measuring snag and tree density at nest and non-sites for white-headed woodpecker in burned forest. All plots consisted of 2 perpendicular 100-m long transects arranged in a plus-shape and centered on the site. Most were 20-m wide, resulting in 0.4 ha plots. Plot widths at Toolbox reflected a measurement protocol developed in green forest, wherein live trees were counted within a narrower plot (6-m wide) to limit logistical demands on surveyors. In burned forest, however, snags are more numerous than live trees, so surveyors at Canyon Creek counted smaller snags (25-50 cm DBH) rather than live trees within a narrower plot.

| Feature | Measurement plot size ‒ m^2^ (length × width) | | |
| --- | --- | --- | --- |
|  | TB | CC | BP |
| Snags 25‒50 m DBH | 4,000 (20 × 200) | 1,200 (6 × 200) | 4,000 (20 × 200) |
| Snags >50 m DBH | 4,000 (20 × 200) | 4,000 (20 × 200) | 4,000 (20 × 200) |
| Trees >25 m DBH | 1,200 (6 × 200) | 4,000 (20 × 200) | 4,000 (20 × 200) |
