# Supplementary material for: Development and evaluation of habitat suitability models for nesting white-headed woodpecker (Dryobates albolarvatus) in burned forest
Source: PLoS One. 2020 May 15;15(5):e0233043. doi: 10.1371/journal.pone.0233043 (PMC7228071; doi:10.1371/journal.pone.0233043)
Supplement: S2 Appendix — (DOCX) [file pone.0233043.s002.docx]

**S2 Appendix.** Area under the receiver-operating-characteristic curve (AUC) for fully parameterized and simplified Maxent models assessing discrimination of nest from available sites in data used to train models at Toolbox, Canyon Creek, and both locations combined. Full models included covariates Slope, Casp, LocBrnOpn, LandBrnOpn, and LandPIPO, whereas simplified models included the subset of these that contributed ≥ 5% of gain for full models.

| Model | AUC for training data | | |
| --- | --- | --- | --- |
|  | Toolbox | Canyon Creek | Combined |
| Full | 0.78 | 0.7 | 0.73 |
| Simplified | 0.79 | 0.7 | 0.72 |
