# Supplementary material for: Development and evaluation of habitat suitability models for nesting white-headed woodpecker (Dryobates albolarvatus) in burned forest
Source: PLoS One. 2020 May 15;15(5):e0233043. doi: 10.1371/journal.pone.0233043 (PMC7228071; doi:10.1371/journal.pone.0233043)
Supplement: S1 Data — (DOCX) [file pone.0233043.s003.docx]

**S1 Data.** Zipped folder containing git repository with all R scripts and R workspace with data compiled needed to replicate all analyses, plots, and tables. This folder also includes three comma-separated text (.csv) files listing all of the candidate weighted logistic regression models and their AIC_c_ values for each of Toolbox, Canyon Creek, and pooled datasets: WLRs_TB.csv, WLRs_CC.csv, and WLRs_TB&CC.csv, respectively. In column 1 of each .csv file, we symbolized covariate as follows:

slope = Slope

cosasp = Casp

brnopn_1ha = LocBrnOpn

brnopn_1km = LandBrnOpn

pipo_1km = LandPIPO

Snag_25_50 = SngMidDens

Snag_ovr50 = SngLrgDens

Tree_ovr25 = TreeDens

PIPO_perc = PIPO%

PIPO = PIPO
