# Supplementary material for: Development and evaluation of habitat suitability models for nesting white-headed woodpecker (Dryobates albolarvatus) in burned forest
Source: PLoS One. 2020 May 15;15(5):e0233043. doi: 10.1371/journal.pone.0233043 (PMC7228071; doi:10.1371/journal.pone.0233043)
Supplement: Attachment — Submitted filename: review2.docx [file pone.0233043.s005.docx]

Excellent and well-written paper. Thanks for the opportunity to review it. This paper will add greatly to our knowledge of white-headed woodpecker use of post-fire landscapes. It will also be useful for forest managers who are faced with increasing fire severity and frequency in the northwestern U.S., where this species is of conservation concern. As the authors point out, managers in these areas are tasked with difficult decisions – salvage logging is done to meet timber targets but can be detrimental to this and other at-risk cavity nesters. Most of my comments were minor or relating to semantics – as well as trying to improve the readability for individuals not familiar with white-headed woodpecker nesting ecology. I commend the authors on a great manuscript and hope this will be published soon, so it can be used by managers.

My biggest concern is that the paper starts out with an emphasis on salvage logging, but in the results and discussion the salvage log results are glossed over because they did not improve the predictive value of the model. To fix this, the authors could re-write the abstract and introduction to remove the focus on salvage logging. Managers need information on salvage logging however, so I’d prefer instead that the authors provide some of the models that did include salvage logging, and/or do more with the descriptive statistics on salvage logging. At the very least, the authors could start the discussion with a paragraph that describes the effects of salvage logging on this species. Even if the paragraph simply states/clarifies that the levels of salvage logging examined in this study had no effect on habitat suitability for white-headed woodpecker.

Specific comments:

Line 61 – suggest changing wording to “managers must strive to identify suitable habitat….”

Line 72 – suggest removing “project”

Line 85-86 – many things can cause geographic variation in model applicability. Suggest adding the following to the beginning of this sentence “Many factors can cause geographic variation in model applicability, however, such as biotic interactions, local adaptation and behavior rules….”

Line 98-99 – soften “require”; suggest replacing with the word “use”, or something similar; not enough is known of this species for such strong language, plus it does not necessarily account for variation in habitat use range-wide

Line 98 – sentence is confusing because it starts out that “white-headed woodpeckers nest in….unburned forested landscapes….. maintained by mixed severity fire”. Do you mean to imply that historically the unburned forests were maintained by mixed severity fire? Otherwise unburned forests being maintained by mixed severity fire seems incongruous. Also note in southern portion of this species range conditions appear different – white-headed woodpecker may not require areas maintained by mixed severity fire in southern CA, for example. Too little research has been done in such locales to be sure either way.

Line 101 – suggest removing “critical”

Line 103 – after ponderosa pine, add “in the northern portion of their range”

Line 104 – remove “suggesting possible source-sink dynamics” or add a sentence to clarify. As written, it is confusing to the reader.

Line 108 – Use of “further” here seems out-of-place. Suggest deleting.

Line 110 – the objectives 1 and 3 are difficult to understand as written. From reading the paper, objective 1 seems to be to develop models of nesting habitat suitability using GIS covariates and field covariates at 3 fires that had some level of salvage logging. Objective 2 is understandable as written. As written, objective 3 seems to be the same as objective 1.

Line 113 – what is meant by the phrase “considering our success in achieving these objectives”? Suggest deleting and start the sentence with “We also evaluated our ability to quantify….”

Table 1 – I appreciate the detailed info in this table, as well as the detailed comments indicated by subscripts.

Line 153 – Use of “affected” is confusing. Suggest stating instead that “Portions of the toolbox and canyon creek fires were salvage logged.” Or clarify early on in this paragraph what is meant by “affected”.

Line 153 – Photos of different fires/study areas – even black-and-white – would be nice to add for the reader.

Line 195 – 35 m is apparently the minimum distance What was the mean and range of distance the non-nest measurements were from the nests? See comment relating to line 300.

Table 2 – Is LandPIPO pre-fire live or post-fire live ponderosa pine?

Line 243 – 25 cm dbh does not seem large to me. Also, in Table 3 large snags are those >50 cm. Suggest deleting the word “large” and simply state that white-headed woodpeckers nest mostly in snags >= 25 cm. I suggest this change be implemented throughout the document. Avoid vague qualifiers “small” or “large” and use numbers (e.g., snags 25-25 cm, > 50 cm, etc).

Table 3, also line 380 – Decay is so vague given the guidelines (yes/no whether ‘nest or center tree showing signs of decay’), plus it wasn’t recorded at one site, so I think it should be removed from the document. It will only confuse the results plus the authors do a nice job on line 252 describing why decay was not modeled. Remove it from the document and simply state that measuring decay is difficult and we did not measure it in this study.

Table 3 – It might be nice to merge tables 2 and 3 so the reader has all the model covariates in one table. I also suggest separating out the covariates that were not included in models. Right now they are indicated with small subscripts. You could have two subheadings: (1) measured covariates that were included in models, and (2) measured covariates that were not included in models.

Line 300 – The weighted logistic model assumes use/nonuse data, in other words? (e.g., Nad’o and Kanuch 2018. Plos one 13:e0200742; rather than use/availability data, like Maxent..) Perhaps this is discussed in detail later, but further consideration of the violations of this assumption are needed here, especially given this is a territorial species and ‘non-use’ sites were apparently as close as 35 m to nests (line 195), which is within an area likely defended by territorial pairs (given that white-headed woodpecker inter-nest/nearest neighbor distances in other studies are typically >100 m). If this species is territorial around nests, it seems it would have been better to buffer around nests before drawing a sample of random points to be used in a use/nonuse design. What if one white-headed woodpecker had a nest in a ‘suitable’ habitat pixel, but it was surrounded by say 2-5 ha of other suitable pixels. Those 2-5 ha of suitable pixels could not have been used by another nesting white-headed woodpecker because of territoriality, and thus may have been incorrectly classified as unsuitable habitat in this design; this may bias the HSIs. Temporal aspects could also be considered – a pair may have used one of the unused pixels the year after you finished the study, even though the suitability for nesting did not change among years. It seems a use/availability SDM is better over all for this study (which is true of most wildlife studies, really). If WLRs are kept in the study to enable analysis of the field-collected variables, please be clear in the text here that you may have violated some assumptions – and be transparent about those assumptions so readers less familiar with SDMs can understand that those results may be a bit more questionable.

Line 303 – I get what the authors are trying to say with this sentence (WLR HSIs primarily quantified relative suitability…) but this argument would need a fair amount of justification – seems more than the scope of this paper. Please consider deleting this sentence.

Line 328 – by “informative”, do you mean transferable?

Line 337 – Why was the proportion of available sites was set to match the proportion of nest sites for the pooled maxent model?

Line 342, and throughout – Especially here in this section describing pooling of study sites, the word ‘location’ gets confusing – just because it has so many meanings. Can the authors use the word “study area” or ’study site’ or ‘fire complex’ consistently throughout the manuscript (and in this section) instead of the word location.

Line 343 – I do not understand this sentence, and thus I do not understand what the ‘hatched’ nests are being used to validate. This paragraph is difficult to follow. (line 343-350) After reading the results it became clear to me that the hatched nests are being used to identify thresholds for low, moderate, and high suitability but I think that has limited value and I suggest deleting the thresholds from the manuscript. See comment for line 481.

Table 4. It is nice to have a table showing the mean values for each covariate.

Line 404 - This statement is unclear to me “supporting a scale-specific tradeoff with burn severity and canopy openness”. Suggest deleting it here, and moving any pertinent argument for or against this concept to the discussion.

Line 462 – Could the authors also present a table with sensitivity and specificity so readers can see in what direction these models performed best (I mean, were the models better at predicting true positives or true negatives?).

Tables 7 and 8. Write out the fire names in the column headings. There are enough acronyms in this table that the reader has to look up without abbreviating the fire names too.

Table 8 - Similarly, suggest removing RS and RS&FC from the first column. Those items are described in the table heading, as well as in the methods, and make the column more confusing than it needs to be. It might be ideal to separate out AUC values for the models transferred to other locations (I mean place those with the subscript “a” in a different table, or in a subheading of this table).

Line 481 – Building off my comment associated with line 343, I do not see the value of the “hatched-nest” analysis. I do think in the methods line 343 the authors could do a better job describing what this analysis is aimed to accomplish. It seems to be used to establish thresholds, but why are thresholds needed? Thresholds are tricky, and are not needed for applying habitat models. They can be quite sensitive – I mean that small changes in the value of thresholds can lead to large changes in the amount of area classified as suitable. Moreover, there are more established ways of assigning thresholds from SDMs like Maxent. I think this analysis should be dropped, and the suitability of habitat from the models be assessed based on the numerical value of the HSIs at given locations. If the authors do choose to keep this analysis, it may help to add it as an objective to the introduction, and please make the methods around line 343 more understandable.

Line 534 – This is well-written.

Line 556 – The species requires snags when breeding so this statement would only hold true if there were snags in the open sites at Barry Point - before the fire.

Line 560 – similar to previous comments, I’d remove the qualifier ‘large’ (and small!) as much as possible. It only adds to confusion among managers about how to provide habitat for this species, which research has shown nests in areas with heavy timber production – thus relatively ‘small’ ponderosa pine trees can also provide nesting and foraging substrates. Best to just use numbers, rather than vague verbiage like large and small.

Line 569 – Use of ‘modulate’ is unclear here.

Line 611 – Poor word choice with ‘favor’. I think the authors mean to imply that prescriptions that protect large decayed snags would benefit white-headed woodpecker. The use of the word favor here at first made me think the authors were suggesting prescriptions should harvest large snags.

Line 616 – This is well written.

Line 625 – How does this sentence relate to white-headed woodpecker? For one thing, nest predators are largely unknown for this species and there is no point in perpetuating theories that are not well-supported by data. Second, the literature indicates that aerial insectivory is not a common foraging method for this species.

General comment – the word mosaic seems a bit overused and is not common parlance. My main reason for commenting is it may confuse readers. Could the authors use the word ‘patchy’?

Line 620 – in general, this section does not add much to the paper. I suggest deleting it.

General comment for the discussion – Two paragraphs would help tie this paper together more nicely: (1) First, start the discussion with a paragraph on the effects of salvage logging, because that is how the abstract starts (and salvage logging is mentioned early in the introduction also). Readers who want to skip the methods and results could then zoom down to the discussion, and the first paragraph would then inform them of the overall effects of salvage logging on this woodpecker species. Even if salvage logging did not impact the habitat suitability, it would still be valuable to start the discussion with a statement to that effect, along with a brief review of the salvage log prescription that were analyzed in this study. (2) add a management implications section and describe implications of results pertaining to salvage logging.
