# Supplementary material for: Development and evaluation of habitat suitability models for nesting white-headed woodpecker (Dryobates albolarvatus) in burned forest
Source: PLoS One. 2020 May 15;15(5):e0233043. doi: 10.1371/journal.pone.0233043 (PMC7228071; doi:10.1371/journal.pone.0233043)
Supplement: Attachment — Submitted filename: ResponseToReview_PlosOne_Feb2020.docx [file pone.0233043.s006.docx]

Author responses are highlighted blue. All line numbers refer to the manuscript Word document with tracked changes.

Associated Editor:

This paper provides an important contribution to the challenge of balancing the needs of native species, such as the white-headed woodpecker, and forest management objectives.  As both of the reviewers point out, the paper is well written but could be improved by some revisions.  Many of the suggestions will increase the utility of the paper and provide additional desirable details.  I will highlight a few of these but I encourage you to read through the reviewers comments carefully as they make very excellent suggestions for ways to improve the manuscript.

One key aspect of this study is the development of HIS models for woodpecker nesting habitat following fire management applied to several novel locations (e.g., transferability).  As the results suggest, it is critical to evaluate whether models can be applied more generally or are highly dependent on local context.  This is a strength of the paper that is not highlighted as well as it could be.  For example, there is no direct comparison of habitat suitability with only remote sensing data only versus with only field-collected data, although only remote sensing data is compared to the combined field and remote sensing data. This omission makes it challenging to assess the value of the data at different scales.  The white-headed woodpecker seems like an excellent model to test this but there may need to be some additional information provided about their ecology to better frame this discussion as Reviewer 1 points out.

We have followed most of Reviewer 1’s recommendations, and justified any deviations from the reviewer’s suggestions below.

I agree with both of the reviewers that the analyses are thorough and, for the most part, well described.  However, there are a few issues highlighted by both reviewers worth noting.  In particular, there are clearly far more models that you describe than are listed in the paper.  It would be very valuable for the reader to be able to evaluate the larger set of candidate models.  As reviewer 2 points out, there is a revised understanding of AIC in the literature that would encourage going beyond the models within 2 AICc units of the top model.  It would also be valuable to provide additional information in Table 6 where you compare the top set of models for each location (e.g., Akaike weights).

We have included complete AIC tables with all candidate models in a supplementary data file for those interested in reviewing them. We have also added a paragraph on the implications and rationale for picking a top model rather than averaging across models to generate predictions in the Discussion.

Reviewer 2 points out that descriptive statistics are mentioned but not described or displayed.

We have added references to our table containing summary statistics to clarify at the locations pointed out by Reviewer 2.

Some other details are mentioned about salvage logging and I agree with reviewer 1 that if that is to be a focus of the paper than more details are needed in both the methods and the results to provide the support for the discussion.  Otherwise, this aspect may need to be de-emphasized.

We provide specific responses to Reviewer 1’s suggestions in this regard below. Salvage logging provides context for the study but was not an explicit part of the analysis and modeling for reasons described in the manuscript. We have followed a general structure advocated for scientific writing wherein we moved from general to specific in the Introduction, and then specific to general in the Discussion. As such, we start and end with the topic of salvage logging.

Both reviewers provide detailed suggestions on the figures and tables that would strengthen the paper (e.g., combining Tables 2 and 3).  Also, more detail is needed in your description of the models, their parameters, and the assumptions for each approach (e.g., reviewer 1 comments specifically on weighted linear regression, reviewer 2 makes suggestions about model evaluation).

We have incorporated most of the reviewers’ suggestions for clarifying our analyses and results, with exceptions noted below.

Additionally, a different set of reference sites are used for each of the models.  It is important to explain why these changes are made and what potential implications they have on the results.

We have added material explaining how and why we left out Barry Point from model development, and use those data solely for evaluating models developed and Toolbox and Canyon Creek.

I agree with reviewer 1 that the hatched-nest analysis is not well described, which undermines its purported value.  Understandably there are a lot of these details that may need to be added to a supplement but they would strengthen the usability of your approach by others.

We have added rationale for relating HSIs with hatched-nest densities and separated this aspect of our work from model evaluation to avoid confusing the two.

The goal of the study is to inform post-fire forest management by identifying suitable habitat for fire-associate species.  You mention the evaluation of management scenarios but this is not demonstrated in the results unless you count the different sites as different scenarios; it would be very valuable to see some example scenarios modeled.  In addition, to explore the issue of transferability the models could be refined with inclusion of a measure of habitat availability, as you provide this a possible explanation for the differences that you found.  Reviewer 1 makes some very constructive suggestions to improve the discussion and better tie the paper together and reviewer 2 highlights some of the elements that need clarification.

We have followed much of the reviewers’ recommendations in this regard. Additionally, we now point to a GIS toolset and accompanying manual that facilitates model application, provides further guidelines, and provides examples with hypothetical management scenarios [62].

Overall, this paper will be a strong contribution to the literature but it needs some revision.  The paper does a very nice job of highlighting two different approaches to modeling habitat suitability while highlighting some of their strengths and weaknesses.  The conclusions are important and seem supported by the data, although some improvement in the presentation will strengthen this connection.

Reviewer 1:

Excellent and well-written paper. Thanks for the opportunity to review it. This paper will add greatly to our knowledge of white-headed woodpecker use of post-fire landscapes. It will also be useful for forest managers who are faced with increasing fire severity and frequency in the northwestern U.S., where this species is of conservation concern. As the authors point out, managers in these areas are tasked with difficult decisions – salvage logging is done to meet timber targets but can be detrimental to this and other at-risk cavity nesters. Most of my comments were minor or relating to semantics – as well as trying to improve the readability for individuals not familiar with white-headed woodpecker nesting ecology. I commend the authors on a great manuscript and hope this will be published soon, so it can be used by managers.

My biggest concern is that the paper starts out with an emphasis on salvage logging, but in the results and discussion the salvage log results are glossed over because they did not improve the predictive value of the model. To fix this, the authors could re-write the abstract and introduction to remove the focus on salvage logging. Managers need information on salvage logging however, so I’d prefer instead that the authors provide some of the models that did include salvage logging, and/or do more with the descriptive statistics on salvage logging. At the very least, the authors could start the discussion with a paragraph that describes the effects of salvage logging on this species. Even if the paragraph simply states/clarifies that the levels of salvage logging examined in this study had no effect on habitat suitability for white-headed woodpecker.

We have revised our paragraph dealing with salvage logging in the Discussion to address the reviewer’s concern (L846-862). We clarify that poor predictive performance with salvage logging covariates does not necessarily imply no relationship, but rather an inconsistency of these relationships that compromises their value for prediction. Given our objectives, we left these relationships out of our models, but we attempt to better describe and connect with other potential studies focused on estimating salvage logging relationships. Considering the importance of salvage logging as a foundational issue for the study, we have retained its emphasis in the abstract and introduction even though it is not explicitly included in our models.

Specific comments:

Line 61 – suggest changing wording to “managers must strive to identify suitable habitat….”

We have instead deleted the referenced sentence. We now refer to the need to identify habitat and predict species distributions together in the subsequent paragraph (L65-68).

Line 72 – suggest removing “project”

Deleted (L81).

Line 85-86 – many things can cause geographic variation in model applicability. Suggest adding the following to the beginning of this sentence “Many factors can cause geographic variation in model applicability, however, such as biotic interactions, local adaptation and behavior rules….”

Adjusted to “Many factors….”

Line 98-99 – soften “require”; suggest replacing with the word “use”, or something similar; not enough is known of this species for such strong language, plus it does not necessarily account for variation in habitat use range-wide

Sentence revised to: “White-headed woodpeckers nest in both recently burned and unburned forest stands typically within landscapes characterized by mosaics of open- and closed-canopy forests often generated and maintained by mixed-severity fire.”

Line 98 – sentence is confusing because it starts out that “white-headed woodpeckers nest in….unburned forested landscapes….. maintained by mixed severity fire”. Do you mean to imply that historically the unburned forests were maintained by mixed severity fire? Otherwise unburned forests being maintained by mixed severity fire seems incongruous. Also note in southern portion of this species range conditions appear different – white-headed woodpecker may not require areas maintained by mixed severity fire in southern CA, for example. Too little research has been done in such locales to be sure either way.

Sentence revised to: “White-headed woodpeckers nest in both recently burned and unburned forest stands typically within landscapes characterized by mosaics of open- and closed-canopy forests often generated and maintained by mixed-severity fire.”

Line 101 – suggest removing “critical”

Deleted as suggested.

Line 103 – after ponderosa pine, add “in the northern portion of their range”

Suggestion implemented.

Line 104 – remove “suggesting possible source-sink dynamics” or add a sentence to clarify. As written, it is confusing to the reader.

Changed to “…suggesting burned forests could represent source habitat for maintaining populations.”

Line 108 – Use of “further” here seems out-of-place. Suggest deleting.

Deleted. This was a holdover from a previous version. Thanks for catching.

Line 110 – the objectives 1 and 3 are difficult to understand as written. From reading the paper, objective 1 seems to be to develop models of nesting habitat suitability using GIS covariates and field covariates at 3 fires that had some level of salvage logging. Objective 2 is understandable as written. As written, objective 3 seems to be the same as objective 1.

Objective 2 was really the means to achieve Objective 3, so we have consolidated 2 & 3 into what is now Objective 2.

Line 113 – what is meant by the phrase “considering our success in achieving these objectives”? Suggest deleting and start the sentence with “We also evaluated our ability to quantify….”

Upon reflection, this sentence did not appear necessary and has been deleted.

Table 1 – I appreciate the detailed info in this table, as well as the detailed comments indicated by subscripts.

Line 153 – Use of “affected” is confusing. Suggest stating instead that “Portions of the toolbox and canyon creek fires were salvage logged.” Or clarify early on in this paragraph what is meant by “affected”.

Revised to “Salvage logging was implemented in portions of the Toolbox and Canyon Creek Fires.”

Line 153 – Photos of different fires/study areas – even black-and-white – would be nice to add for the reader.

Unfortunately, we only have photos for one of the three wildfire locations – Canyon Creek. We are happy to add these, but we did not do so in this draft because it seems like the reviewer’s intention is to allow the reader to compare photos of different locations, which we cannot support with photos from just one location.

Line 195 – 35 m is apparently the minimum distance What was the mean and range of distance the non-nest measurements were from the nests? See comment relating to line 300.

We have clarified to state that “…nests were never located within 30 m of non-nest sites (resolution of remotely sensed data) during the study period.” The minimum distance should be sufficient to support this statement.

Table 2 – Is LandPIPO pre-fire live or post-fire live ponderosa pine?

We have added the clarifier “pre-fire” to the description in Table 2.

Line 243 – 25 cm dbh does not seem large to me. Also, in Table 3 large snags are those >50 cm. Suggest deleting the word “large” and simply state that white-headed woodpeckers nest mostly in snags >= 25 cm. I suggest this change be implemented throughout the document. Avoid vague qualifiers “small” or “large” and use numbers (e.g., snags 25-25 cm, > 50 cm, etc).

We have specified the DBH cutoff and dropped the “large” qualifier everywhere that we refer to the minimum size for snags to be considered a potential nest site. We have also examined all uses of the words “small” and “large” to ensure there is no potential confusion and that their use is appropriate given the context.

Table 3, also line 380 – Decay is so vague given the guidelines (yes/no whether ‘nest or center tree showing signs of decay’), plus it wasn’t recorded at one site, so I think it should be removed from the document. It will only confuse the results plus the authors do a nice job on line 252 describing why decay was not modeled. Remove it from the document and simply state that measuring decay is difficult and we did not measure it in this study.

We have removed this variable from the paper as requested and instead rely solely on references to support discussion regarding decay.

Table 3 – It might be nice to merge tables 2 and 3 so the reader has all the model covariates in one table. I also suggest separating out the covariates that were not included in models. Right now they are indicated with small subscripts. You could have two subheadings: (1) measured covariates that were included in models, and (2) measured covariates that were not included in models.

The two tables have been combined as requested into the revised Table 2.

Line 300 – The weighted logistic model assumes use/nonuse data, in other words? (e.g., Nad’o and Kanuch 2018. Plos one 13:e0200742; rather than use/availability data, like Maxent..) Perhaps this is discussed in detail later, but further consideration of the violations of this assumption are needed here, especially given this is a territorial species and ‘non-use’ sites were apparently as close as 35 m to nests (line 195), which is within an area likely defended by territorial pairs (given that white-headed woodpecker inter-nest/nearest neighbor distances in other studies are typically >100 m). If this species is territorial around nests, it seems it would have been better to buffer around nests before drawing a sample of random points to be used in a use/nonuse design. What if one white-headed woodpecker had a nest in a ‘suitable’ habitat pixel, but it was surrounded by say 2-5 ha of other suitable pixels. Those 2-5 ha of suitable pixels could not have been used by another nesting white-headed woodpecker because of territoriality, and thus may have been incorrectly classified as unsuitable habitat in this design; this may bias the HSIs. Temporal aspects could also be considered – a pair may have used one of the unused pixels the year after you finished the study, even though the suitability for nesting did not change among years. It seems a use/availability SDM is better over all for this study (which is true of most wildlife studies, really). If WLRs are kept in the study to enable analysis of the field-collected variables, please be clear in the text here that you may have violated some assumptions – and be transparent about those assumptions so readers less familiar with SDMs can understand that those results may be a bit more questionable.

We have clarified to state “…we are reasonably confident that nests were never located within 30 m of non-nest sites (resolution of remotely sensed data) during the study period.” In principle, our models do not assume that non-nest sites were never used for any activities associated with nesting, as they certainly could occur within the 1-km radius buffer used for landscape scale variables in our models. We simply assume strictly speaking that non-nest sites never contained an actual nest at the minimum resolution (30m) of our environmental data during our study.

Line 303 – I get what the authors are trying to say with this sentence (WLR HSIs primarily quantified relative suitability…) but this argument would need a fair amount of justification – seems more than the scope of this paper. Please consider deleting this sentence.

In the interest of informing interpretation, we think it is important to clarify what HSIs represent in terms of underlying process. We have revised this sentence to more clearly acknowledge the ambiguity in underlying process without making claims that require justification (L396-398).

Line 328 – by “informative”, do you mean transferable?

We have clarified to “…useful for discriminating nest from reference sites…”.

Line 337 – Why was the proportion of available sites was set to match the proportion of nest sites for the pooled maxent model?

The explanation is provided by the preceding sentence. To clarify, we have rephrased the preceding sentence to “So that pooled models would be informed equally by each individual wildfire location, we adjusted the analyzed data as follows.” Additionally, we deleted the last sentence of the paragraph as it is redundant with this one.

Line 342, and throughout – Especially here in this section describing pooling of study sites, the word ‘location’ gets confusing – just because it has so many meanings. Can the authors use the word “study area” or ’study site’ or ‘fire complex’ consistently throughout the manuscript (and in this section) instead of the word location.

We looked through all uses of the term “location” and added adjectives “study” or “wildfire” where helpful to clarify. We are now careful throughout to only use this term to refer to individual forested areas burned by wildfire. In fact, when reviewing our use of this term, we did find a couple instances where we mistakenly referred to “nest locations” and corrected these to “nest sites”. We do not see how our terminology scheme is any more difficult to follow than others suggested by the reviewer. We specifically avoid study site to avoid confusion with nest sites. The current scheme is also consistent with terminology used in other related publications produced by our group [e.g., 32].

Line 343 – I do not understand this sentence, and thus I do not understand what the ‘hatched’ nests are being used to validate. This paragraph is difficult to follow. (line 343-350) After reading the results it became clear to me that the hatched nests are being used to identify thresholds for low, moderate, and high suitability but I think that has limited value and I suggest deleting the thresholds from the manuscript. See comment for line 481.

Strictly speaking, relationships with hatched nest densities are not intended for model evaluation *per se*, but rather to aid interpretation and application for informing management (*HSI relationships with hatched-nest densities*). We have therefore separated this portion of the methods into a separate sub-section. From our work with forest managers, they often want to identify suitability classes to inform their planning. We therefore think suggesting potential categories based on the HSI relationship with hatched nest densities will be highly desirable. Additionally, this helps with application to inform population objectives (e.g., how much habitat is needed to support a target number of breeding individuals. We have in fact received positive feedback on this aspect of our presentation of HSI models.

Table 4. It is nice to have a table showing the mean values for each covariate.

Line 404 - This statement is unclear to me “supporting a scale-specific tradeoff with burn severity and canopy openness”. Suggest deleting it here, and moving any pertinent argument for or against this concept to the discussion.

The referenced phrase has been deleted as it is covered in the Discussion.

Line 462 – Could the authors also present a table with sensitivity and specificity so readers can see in what direction these models performed best (I mean, were the models better at predicting true positives or true negatives?).

Sensitivity and specificity are threshold-dependent metrics, so this comment conflicts with the reviewer’s earlier suggestion to remove evaluation of thresholds from the manuscript. Regardless, classification error rates would not be readily interpretable given the relative nature of HSIs and the weighted nature of the data to which our models have been fitted. Instead, we provide estimates of hatched-nest densities both in continuous and categorical form. Relationships with hatched-nest densities are much more informative than sensitivity and specificity values could be, so we do not see value in adding to the already large number of tables to present sensitivity and specificity.

Tables 7 and 8. Write out the fire names in the column headings. There are enough acronyms in this table that the reader has to look up without abbreviating the fire names too.

Done.

Table 8 - Similarly, suggest removing RS and RS&FC from the first column. Those items are described in the table heading, as well as in the methods, and make the column more confusing than it needs to be. It might be ideal to separate out AUC values for the models transferred to other locations (I mean place those with the subscript “a” in a different table, or in a subheading of this table).

The acronyms have been removed as suggested. We did not separate AUCs representing transference to alternate locations because we see presentation of these types of values in this format as relatively standard (e.g., confusion or contingency tables), and we think separation would unnecessarily complicate the information presented.

Line 481 – Building off my comment associated with line 343, I do not see the value of the “hatched-nest” analysis. I do think in the methods line 343 the authors could do a better job describing what this analysis is aimed to accomplish. It seems to be used to establish thresholds, but why are thresholds needed? Thresholds are tricky, and are not needed for applying habitat models. They can be quite sensitive – I mean that small changes in the value of thresholds can lead to large changes in the amount of area classified as suitable. Moreover, there are more established ways of assigning thresholds from SDMs like Maxent. I think this analysis should be dropped, and the suitability of habitat from the models be assessed based on the numerical value of the HSIs at given locations. If the authors do choose to keep this analysis, it may help to add it as an objective to the introduction, and please make the methods around line 343 more understandable.

We have clarified the purpose of this analysis. Additionally, we separated this analysis from evaluation with AUC in the methods to avoid placing too much weight on these relationships for evaluating models, although we note that Hirzel [53] and subsequent authors would probably consider the monotonicity of these relationships to inform evaluation. The use of plots that relate intensity of habitat use (nest densities here) with HSIs to identify classification thresholds is described by Hirzel [53]. We see this as more straightforward than other approaches suggested in the literature for delineating thresholds (e.g., formulas involving sensitivity and specificity) and more readily interpretable for informing forest management (e.g., population targets).

Line 534 – This is well-written.

Thank you.

Line 556 – The species requires snags when breeding so this statement would only hold true if there were snags in the open sites at Barry Point - before the fire.

We have reworded to “These open-canopy sites were present prior to wildfire, so assuming availability of snags at these sites, white-headed woodpeckers at Barry Point may not have depended as heavily on wildfire to generate canopy openings desirable for nesting.”

Line 560 – similar to previous comments, I’d remove the qualifier ‘large’ (and small!) as much as possible. It only adds to confusion among managers about how to provide habitat for this species, which research has shown nests in areas with heavy timber production – thus relatively ‘small’ ponderosa pine trees can also provide nesting and foraging substrates. Best to just use numbers, rather than vague verbiage like large and small.

We have added DBH ≥ 25 cm in parentheses to clarify. We nevertheless retain the “large” descriptor. To our understanding, large ponderosa pine are of greater value as resources for nesting and foraging, and we think the weight of evidence thus far supports placing value on retention of relatively large trees as resources for nesting. Just because WHWO are capable of using smaller trees, doesn’t mean smaller trees are of equal value as larger trees. Although we don’t want to discount the potential value of smaller trees where larger ones are unavailable, we do not want to imply that prescriptions that only retain smaller trees support habitat conservation as well as those that explicitly retain larger trees. Unless and until the weight of evidence indicated tree diameter is not important at all for nest site suitability, we will continue to retain “large” to describe snag suitability for nesting.

Line 569 – Use of ‘modulate’ is unclear here.

We have reworded to “Models may need to allow relationships with slope and percent ponderosa pine to be modulated by habitat availability to correctly inform predictions.” The reference provided also explains the concept in more depth for interested readers.

Line 611 – Poor word choice with ‘favor’. I think the authors mean to imply that prescriptions that protect large decayed snags would benefit white-headed woodpecker. The use of the word favor here at first made me think the authors were suggesting prescriptions should harvest large snags.

Good point. We have changed to “…retain relatively large snags….”

Line 616 – This is well written.

Thank you.

Line 625 – How does this sentence relate to white-headed woodpecker? For one thing, nest predators are largely unknown for this species and there is no point in perpetuating theories that are not well-supported by data. Second, the literature indicates that aerial insectivory is not a common foraging method for this species.

The reviewer uses the wording “not well-supported” but rather seems to be implying that this a zombie hypothesis for which existing evidence has effectively debunked and is therefore not worth considering. We agree that this hypothesis is not clearly supported by the data, but disagree that there is sufficient evidence to ignore it, which is why we suggest it as possible and worth considering for future research. We have added a reference documenting the potential importance of red squirrels as nest predators in conifer forests. Additionally, we provide references describing their potential role in shaping cavity-nesting bird associations with burned forests. The reviewer also appears to suggest that nest predation is not important for WHWO when in fact Wightman [27] clearly report that predation was the main cause of nest failure in burned forests. Additionally, we point out that even if nest failure rates are low, predators may nevertheless shape nesting ecology. WHWO nest site selection may effectively avoid nest predation by selecting low-risk sites, which potentially could include open-canopy sites generated by wildfire.

General comment – the word mosaic seems a bit overused and is not common parlance. My main reason for commenting is it may confuse readers. Could the authors use the word ‘patchy’?

We do not see how the term “patchy” could replace “mosaic” in the context of this manuscript. We also do not see how this term could be interpreted incorrectly given our introduction of the concept early in the manuscript (L113-118). Finally, even if this term is not “common parlance,” our group has established a precedent for using this term in previous publications [22, 34].

Line 620 – in general, this section does not add much to the paper. I suggest deleting it.

General comment for the discussion – Two paragraphs would help tie this paper together more nicely: (1) First, start the discussion with a paragraph on the effects of salvage logging, because that is how the abstract starts (and salvage logging is mentioned early in the introduction also). Readers who want to skip the methods and results could then zoom down to the discussion, and the first paragraph would then inform them of the overall effects of salvage logging on this woodpecker species. Even if salvage logging did not impact the habitat suitability, it would still be valuable to start the discussion with a statement to that effect, along with a brief review of the salvage log prescription that were analyzed in this study. (2) add a management implications section and describe implications of results pertaining to salvage logging.

We strongly disagree with this suggestion. The referenced paragraph suggested for deletion provides substantive and relevant material for guiding future research towards developing our understanding of nesting habitat for WHWO and predicting their nesting distributions, which is entirely appropriate for a manuscript with our stated objectives. Reviewer 2 does not appear to dispute the relevance of this paragraph, and Reviewer 1 does not provide any rationale for removing it, so we have retained it. Second, beginning the discussion with salvage logging would be inappropriate because we did not estimate relationships with salvage logging. Salvage logging and post-fire forest management together provide important context compelling the implementation of this study, which is why we begin with ideas on salvage logging. Nevertheless, our objectives concerned the development of predictive models, and the first paragraph of the Discussion (in our view) should describe major conclusions arising from our analysis and the extent to which we met our objectives. We follow an hourglass structure generally advocated for scientific writing, whereby we move from general to specific in the Introduction, and then specific to general in the Discussion. Following this structure, starting and ending with salvage logging makes the most sense, which is why the final paragraph discusses implications for salvage logging and post-fire forest management. We do not see how we could get to this final point of Discussion without first establishing major conclusions with respect to the value of our models for prediction.

Reviewer 2:

The authors have submitted a well-written manuscript that reports on the use of Maxent to make predictions about habitat suitability for White-headed Woodpeckers at burns in Oregon, USA. The basic approach was to compare nests vs non-nest sites at three burns. Two of the sites appeared to have better predictive ability but the third site differed, making any transferability inappropriate.

I really liked the manuscript and appreciate the depth of analyses that went into the patterns reported in the manuscript. Coupled with the extensive field data, this manuscript represents a lot of effort! My comments are mainly editorial in nature.

The main issue I have is with the dumbing down of quantitative analyses. Rather than telling the reader that previous analyses didn’t show any difference so we only present the simplified version, the authors should provide all of the information and let the reader decide for themselves. Likewise, if the authors are going to discuss analyses that were conducted, they need to present those analyses. And, finally, if the authors are going to rank candidate models, show the reader ALL of the models rathe than filtering it down to just the delta AICc < 2. I find this particularly annoying.

We have followed the reviewer’s recommendations by adding requested information where possible, including complete AIC tables and AUCs for Maxent models in a new appendix. We could not, however, include models fitted at Barry Point as this line of analysis was abandoned before it was completed once it became clear that it would not be informative. We have adjusted our language to make this step in our analysis clearer, however.

Here are more specific comments on this issue:

130 More details need to be provided on this “preliminary analysis”. What is meant by “substantially different relationships”?

We appreciate the reviewer’s desire to see results rather than taking us at our word, but we never fully completed the development of Barry Point models and their application at the other two locations. Once we saw the initial Maxent models, we abandoned further model development at Barry Point (we never ran WLR models). We are confident that the ultimate conclusion that Barry Point differs from the other two locations would not change by presenting Barry Point models, and because of the limited sample size at Barry Point, we do not think relationships observed there could contribute confidently and meaningfully (without additional data) to our understanding of species nesting ecology. We therefore decided it wasn’t going to be worth our time, manuscript space, nor the attention of readers to follow through on Barry Point models, and at this point it is beyond our capacity to go back and do so. We think covariate summary statistics and the consistently poor performance of models developed at Canyon Creek and Toolbox when applied at Barry Point, are sufficient to document the limitations of model prediction to Barry Point, along with the need for more data to develop models useful for other parts of the species range. We have elaborated further on our thinking in the revised version of the manuscript (L151-175).

288-290 Is this statement about verifying comparable performance with more complex models documented anywhere? The reference to citation 35 pertains to a 2015 paper comparing nest use HSIs, with presumably an entirely different database. I think this information should be included in an appendix.

We have added and now refer to Appendix S2, and we have removed reference to published studies here (L379-381).

311-312 Is this statement about only using first-order linear covariate relationships related to the previous statement about using simpler models? As before, this information should be provided in a supplement / appendix.

We have reworded this sentence to clarify (L381-383).

366 I did not have access to S2 data so was unable to evaluate or provide feedback. Likewise, at line 851, the authors indicate there is a zipped folder with a git repository. None of this was available when evaluating this manuscript.

We have compiled and included the zip file with R scripts and workspace.

414 The authors compare the global to a reduced subset of models. I thought the authors previously said they did not use more complex models. Please clarify. At the very least, the reader needs to know what models were in the global models vs the reduced models if the authors are going to reference global vs reduced in Table 5.

We had included the global models in this table to show the variables with less than 5% contributions that were cut out in the reduced models, but for the sake of clarity, we have removed reference to global models from the table and just show the models that were ultimately retained for evaluation (Table 4).

439 The authors present only those candidate models within 2 AICc units from the top model. The full suite of models should be provided in a supplement / appendix. The reader would be interested in knowing how many models were withing 2-3 AICc units. Was there a clear break or was there a continuum of supported models that extended beyond 2 units? This dumbing down of the data presentation can be particularly annoying to readers that want to see the full suite of candidate models.

We have included files listing all candidate models in S3 Data.

452 Most modern day information theorists recommend abandoning the delta < 2 mantra. See extensive discussion of this by Burnham et al. (2011). They advocate against that arbitrary cutoff. My recommendation to the authors is to adopt a more modern approach where any of the models with AICc<10 are considered. The obvious solution would be to use model-averaging instead of only relying on the <2 models.
Here is what Burnham et al. (2011) write:
Δ>2 Rule. Some of the early literature suggested that models were poor (relative to the best model), and might be dismissed if they had Δ>2. This arbitrary cutoff rule is now known to be poor, in general. Models where Δ is in the 2–7 range have some support and should rarely be dismissed. Inference can be better based on the model likelihoods, probabilities, and evidence ratios and, in general, based on all the models in the set. From these quantitative measures one can then assign their own value judgment if they wish.
Burnham, K. P., D. R. Anderson, and K. P. Huyvaert. 2011. AIC model selection and multimodel inference in behavioral ecology: Some background, observations, and comparisons. Behavioral Ecology and Sociobiology 65:23-35.

We do not use the Δ>2 cutoff for anything other than deciding which models to present in Table 5. Rather than implementing model-averaging, we have opted to select a single top model that represents the best apparent balance of fit and parsimony (according to AIC) as now explained in a new paragraph in the Discussion (L952-961).

573 Like I have mentioned previously, details about analyses used need to be documented. What are these “descriptive statistics” and where can they be found? Similarly, lines 610-611 discuss “descriptive statistics” about “logging prescriptions” favoring large decayed snags.

We have added references to Table 3 in both locations.

Line Edits

20 Insert scientific name for the woodpeckers

Done.

35 AUC is not defined; likewise it is not defined in the main text (line 275) at first mention. Instead, it is defined all the way down at 325.

Definitions added.

36 Add two decimals to 57.00 so format matches 0.53

It is actually supposed to be 0.57. Thanks for catching.

90 Define the term “transferability”

Definition added.

127 Insert “(hereafter Canyon Creek Fire;”

Done.

134 The sc name was already introduced at line 103.

Second instance deleted.

135 The authors are inconsistent on whether they introduce e.g. or i.e. within parentheses or with merely a comma. This problem occurs throughout.

We have now placed all within parentheses or brackets.

137 All numbers in the thousands should have a comma for consistency. The authors arbitrarily include the comma or not. See 1,603; 4,347; 4,727; and subscript e for example in Table 1. See also line 358 among other places.

We have scanned through the document and added commas to numbers of 1,000 or more where we found them.

165 Should be “prior to”

Changed to “before”.

168 This is the first mention of DBH so it should be “diameter at breast height (DBH) > 23 cm per hectare”. The authors introduce the abbreviation before the definition.

Corrected.

204 Add extra hyphen “30-m-pixel”

Added.

218 The authors need to provide a citation for the statement about the woodpeckers nesting in canopy openings.

References added (L268).

266 Typo “diamter”

Corrected.

327 The statement that AUC=1 indicates perfect discrimination needs a citation.

Added.

328 This line is the first occurrence of “confidence interval” so it should be abbreviated here (not later in the ms at line 354 as it is currently formatted).

Corrected.

344 This is the first introduction of “hatched nests” so the “(i.e. nests with at least 1 nestling)” information should appear here, not on line 347.

Corrected.

346 The authors indicate that by using hatched nests, detection probability was nearly perfect. How does this approach account for nests that failed before hatching? Does this not bias towards habitat with successful nests (e.g. those areas with less predators, unsuitable nest trees, etc).

We have added additional rationale for relationships with hatched-nest densities that should address this concern (L466-469). Basically, we are trying to anchor interpretation of the HSIs to some concrete metric related to habitat suitability. Fitness is an important component of habitat suitability, and hatching success is a component of fitness.

352 Two nests were excluded plus any other nests that went undetected, of course.

Indeed, but we have already pointed out that hatched nests are highly detectable, so we are assuming we missed a negligible number of hatched nests.

414 This is the first time the abbreviations TB and CC have been used. They need to be defined before the abbreviation can be introduced like this in the table.

We have removed this abbreviation.

456 At some point, the authors will want to clean up their tables so that the numbers are aligned by decimal point. I appreciate that they may not have done this for the submitted version of their manuscript.

This seems like a production issue rather than an editorial one. We are happy to do this if requested by the journal.

470 Remove hyphen in bootstrapped so it matches the style used throughout the ms.

Corrected.

559 Unclear whether this 40% figure comes from the literature or is based on unpublished data. Either way, the authors need to provide a citation (if published) or a sample size that this percentage is based on if this is unpublished data.

Sample size added.

570 Change “correct” to “correctly”

Corrected.

581 Unclear what levels are being referenced here. Levels of what?

The sentence has been revised in a way that should clarify (L860).

594 Are the authors speaking more generally here or are they specifically referring to WHWOs when they mention “habitat reserves”?

Clarified that reserves would be for white-headed woodpecker.

598 Insert hyphen “hatched-nest densities”

Corrected.

600 Is this objective of reaching “particular population targets” in play? If so, a citation and / or more background information should be provided here.

At this point, we are discussing the general idea. We do not have particular published references describing cases where forest managers have developed management plans with population targets. We do, however, now provide a reference that includes demonstrations of potential applications [62 referenced in Discussion].

610 Unclear what this 40% number represents. How is “dominated or co-dominated by ponderosa pine” defined? Does one just count up all of the trees within a 1-km radius and calculate what proportion of trees are PP? If so, how does one define co-dominated? Some folks are not as up on forestry lingo and need a bit of help understanding the terminology.

We added a footnote to Table 2 clarifying that ponderosa pine forest for LandPIPO was classified following GNN data along with a reference. We also corrected the formatting of references to data sources providing forest structure and composition [42] and burn severity [41] to integrate with the numbered formatting to correspond with the rest of our references and Plos One format.

617 Are the authors advocating for habitat-fitness data as a prerequisite for constructing HSI models, or just that it would be nice to have? Ambiguous as worded.

Changed wording to state that habitat-fitness relationships would complement HSI models to inform management.

641 Citation needed for the statement about restoring and maintaining forests and the disruption by humans.

References added.

648 These geographic terms (e.g. East Cascades, Blue Mountains, Modoc Plateau, North Cascades) will mean nothing to a casual reader. Please provide geographic reference to put in context. Oregon? USA?

We have added the parenthetic note “(northwestern U.S.A.)”. Additionally, we do establish in the Introduction that white-headed woodpeckers are endemic to western North America.
