# Supplementary material for: Development and evaluation of habitat suitability models for nesting white-headed woodpecker (Dryobates albolarvatus) in burned forest
Source: PLoS One. 2020 May 15;15(5):e0233043. doi: 10.1371/journal.pone.0233043 (PMC7228071; doi:10.1371/journal.pone.0233043)
Supplement: Attachment — Submitted filename: ResponseToReview_PlosOne_Apr2020.docx [file pone.0233043.s007.docx]

Author responses are highlighted blue. All line numbers refer to the manuscript Word document with tracked changes.

Associated Editor:

This paper provides an important contribution to the challenge of balancing the needs of native species, such as the white-headed woodpecker, and forest management objectives and needs only minor revisions.  I appreciate your thorough attention to the suggestions and questions of the initial reviewers.  As a result of these changes, the paper is much stronger, better written and provides more compelling conclusions.  The revised methods have improved clarity and better highlight the important aspects of the approach utilized.  I appreciate the revision of the analysis to address issues mentioned by the reviewers and the results now better support the conclusions. The discussion provides an excellent overview of the results in a broader context and makes valuable suggestions on future research.  Reviewer #2 has some minor suggestions that you should address in your revisions.  Please, note, though, that Reviewer #3 has highlighted some concerns about the analytical methods leading to their decision to reject.  You will certainly need to address the issue of quadratic effects and provide clarification about why that might not be appropriate or feasible for these data and/or the application intended.  While I agree that it might be worthwhile to include some additional landscape analyses to further explore configuration effects, there are a seemingly endless number of variables you could include in HSI models.  However, the suggested analyses (e.g., fragstats) do not seem necessary if the potential limitations in the conclusions you can make with the current analysis are explicitly acknowledged in the discussion and you think these additions are beyond the scope of the study.  Overall, this paper will make a strong contribution to the field with some additional minor revisions.

Reviewer #2:

I was the proverbial reviewer 2 in the previous version of this manuscript. I have read over the new version of the manuscript and reviewed all of the line-by-line responses to my previous comments. As I said previously, this is a great manuscript and well written. The authors have done a great job revising the manuscript. I thank them for their thorough treatment of the reviewer comments. Well done!

We thank the reviewer.

Only a couple of minor remaining edits:

85 Change “Due to” to “Because of”. Due to modifies a noun, it is incorrect to use it to modify a verb.

Corrected, although “funding limitations” functions as a noun rather than a verb

164 The authors might consider using “autumn” instead of “fall”. The latter term is only used in North America.

Corrected.

172 Redundant “diameter at breast height (DBH)” with previous line.

Corrected.

360 Inconsistent missing comma in 5,000

Comma added.

368 Should this be “S3 Appendix” to match the terminology used for S1 and S2, previously? See also line 441.

We have in fact entitled this supplement “S3 Data” following PlosOne author guidelines, so we have left the in-text reference as is.

503 Insert hyphen “field-collected”

Corrected.

506 You need a “respectively” after the statement about large circles are density estimates and error bars are 95% CIs.

Added.

Reviewer #3:

The authors have a well-written paper about a timely subject. The choice of topics and the broad experimental design seem appropriate to answer their research questions. A red flag I noticed in the methods is that the authors didn’t consider quadratic effects. Considering burn intensity and tree density likely have some optimum values which are neither 0 nor 100%. It seems like there is a strong a priori justification for examining quadratic effects. This choice absolutely needs to be justified. For example white-headed woodpeckers have a negative relationship with tree density, but clearly it cannot have a maximum at 0, as there would be no trees for nesting.

We have added material to the discussion to acknowledge the potential for examining quadratic relationships in future research (L652-656). Additionally in response to this specific comment, the reviewer appears to be conflating burn severity and tree density. 100% severity does not imply 0 trees for nesting remaining on the landscape. In fact, because this and other disturbance-associated woodpeckers require standing dead (not live) trees for nesting, a forest stand may suffer 100% mortality and be maximally desirable for nesting as long as a sufficient number of trees are not burned to the ground, which is typically the case. Therefore, the requirement of (dead) trees for nesting does not suggest a possible quadratic effect with burn severity. One further point to make is that we have restricted our study to forested landscapes. By restricting our sampling to areas with at least some trees, the quadratic relationship expected by the reviewer may not be represented in our data or in similar datasets.

A second red flag is that you argue landscape configuration is likely interacting with forest characteristics to influence results lines 550-587. Why not use fragstats or some other approach to quantifying landscape configuration and incorporate these characteristics among the sites into the modeling approach?

We have added a sentence suggesting this possibility for future research (L563-565).

I was thinking about these puzzling methodological choices so I ran supplied example code. The modeling used was more appropriate for an exploratory data analysis, but seems like a pretty flagrant abuse of data dredging best practices. Considering the obvious ignoring of appropriate a priori hypotheses, and instead building gigantic linear combinations of model covariates via a data dredging approach, the authors may benefit from reexamining the methodological tactics employed here.

We strongly disagree that our approach represents flagrant data dredging. Restricting candidate models that represent *a priori* hypotheses does not represent the only valid approach to analyses. We have provided an additional paragraph to provide context for our approach in the broader landscape of ecological research (L598-604).
